# Improving access to family planning services through community pharmacies: Experience from The Challenge Initiative in three counties in Kenya

**DOI:** 10.3389/fgwh.2023.1060832

**Published:** 2023-04-24

**Authors:** Nancy Aloo, Paul Nyachae, Njeri Mbugua, Morine Sirera, Kenneth Owino, Peter Kagwe, Njeri Nyamu, Mohammed Hanif, Miriam Ndirangu

**Affiliations:** ^1^ The Challenge Initiative, East Africa Hub, Jhpiego, Nairobi, Kenya; ^2^ Ministry of Health, Mombasa, Kenya; ^3^ Kenya Pharmaceutical Association, Nairobi, Kenya

**Keywords:** family planning (FP), pharmacies and drug stores, Kenya, The Challenge Initiative, improving access to care

## Abstract

Pharmacies play a vital role in improving access to family planning (FP) services in urban areas. They complement the resource-limited public health system and are viewed as key access points for contraceptives among young people (10–24 years) and the general population. The Challenge Initiative East Africa (TCI EA), in collaboration with the health management teams of Mombasa, Kilifi, and the Nairobi counties in Kenya and the Kenya Pharmaceutical Association (KPA) piloted an innovative public-private partnership (PPP) engagement to improve access to quality FP services offered at pharmacies in urban areas. The pilot project built the capacity of pharmacists, strengthened the referral system to public health facilities, and made FP data accessible and visible to drive informed decision-making. This paper describes the strategies employed and the outcomes. The initiative targeted 150 pharmacies across the three counties from June 2019 to December 2020 period. Our assessment shows that this intervention delivered FP commodities to 43,632 FP client visits; 71% for female clients and 21% for males. Adjusting for couple years of protection and seasonality, this translates to about 2,800 annual FP clients obtaining modern contraception in a 12-month period, including 48% injectables, 25% oral contraception, 24% emergency contraception, and 3% condoms. The majority of clients (75%) were older than 24 years, 21% were 20–24 years, 3% were 15–19 years, and 1% were less than 15 years. In addition, 327 clients were referred to a public sector facility for other methods. This intervention demonstrates the potential of pharmacies in contributing to FP uptake and provides a framework for improving access to quality FP services by pharmacies. There is potential to scale such an approach beyond the 3 counties, given the involvement and reach of KPA and the Ministry of Health (MoH) health management teams.

## 1. Introduction

Women of reproductive age in Kenya continue to face a high unmet need for family planning. 26% of women have an unmet need for contraception and this is highest among adolescents and those aged 20–29 years (unmet need at 30%) compared to the older ones (at 22% for those aged 30–34 years and 25% among those aged 34–44 years) (1). Various factors affect access to modern contraception including long distances to service points, myths and misconceptions affecting uptake, the fear of side effects from use, and lack of social support among other factors. Public health facilities serve 60% of users, while the private sector accounts for 34%—mainly private hospitals/clinics (21%) and community pharmacies (10%) (2). Expanding access points is thus a potential route through which more women needing contraception in Kenya could access it, which would in turn help meet the national FP2030 goals aiming to reduce the access barriers to modern contraception (FP2030) (1). Some of the other potential service delivery points that the Government of Kenya proposed to use in the FP2020 agenda include community pharmacies and private health facilities.

Community pharmacists serve as an important addition to increasing access to primary healthcare services and supporting the resource-limited public health system as they are viewed as the key source for access to contraceptives and services for young people (10–24 years) where the highest unmet need is reported (FP2030) (3), as well as the first point of contact for women and the general population for medicine (4). Youth unwilling or unable to go to a health facility seek out community pharmacies for the provision of contraception—that is, over-the-counter (openly accessible at a pharmacy), or behind-the-counter (dispensing contingent on evaluation from a pharmacist). The provision of contraceptives at the community pharmacy has been shown to help overcome barriers to access among young people (5) who face various barriers to accessing contraception (6).

In Kenya, community pharmacies are authorized to offer select family planning (FP) services, such as female and male condoms, oral contraceptives, emergency contraceptives, and injectable intramuscular and subcutaneous depot medroxyprogesterone acetate (DMPA) (2). They also counsel and refer clients to health facilities for other FP services not available within the community pharmacies (7). To offer these services effectively, pharmacists are required to have the competency to dispense contraceptives and provide method specific counseling on all available contraceptive methods. This entails explaining how all methods work and the possible side effects, while informing the potential users of the different methods available to them based on their desire to either prevent or delay a pregnancy. Locally known as “chemists” in Kenya, pharmacies are privately owned, regulated, and inspected by the Pharmacy and Poisons Board (PPB). They are also recognized as retail drug outlets by the MoH and affiliated with professional organizations, such as the Kenya Pharmaceutical Association (KPA) which provides capacity strengthening and collective bargaining support. One in ten FP clients in Kenya obtains contraception method of choice from community pharmacies (8, 9).

Although community pharmacies have been recognized in many countries, including Kenya, for their potential to improve health indicators (4, 10), they are largely missing from the countries' health strategies, policies and regulations, and monitoring. Consequently, there is little information about the type and quality of information and services that they offer. There are concerns that community pharmacies may do little to provide re-supply reminders to clients buying methods, such as pills and injectables, for example. They may not refer women methods they do not sell since they are not usually integrated into the larger public health system, and they do not have an economic incentive to do so (11). In Kenya, it is difficult to measure service utilization due to a lack of coordinated mechanisms from the government for data collection and analysis of clients seeking services from private pharmacies, thus contributing to poor data visibility on the actual impact arising from this sector.

The primary aim of this paper is to describe The Challenge Initiative's unique engagement with the Kenya Pharmaceutical Association (KPA) to improve access to FP services through community pharmacies in Kilifi, Nairobi, and Mombasa counties in Kenya. Specifically, the paper seeks to highlight the effects of the strategies employed by TCI-supported public-private partnership (PPP) and to share learnings from the implementation of the adaptations by different implementers and stakeholders interested in public-private partnerships. By reviewing and evaluating findings from the implementation of this intervention, we aim to contribute to the evidence base on how best to improve access to FP services through community pharmacies.

## 2. Context

### 2.1. Program—The Challenge Initiative

Funded by the Bill & Melinda Gates Foundation, Bayer AG, Comic Relief Foundation in the United Kingdom, and private philanthropists, TCI empowers cities to rapidly and sustainably scale up family planning and adolescent and youth sexual and reproductive health (AYSRH) high-impact interventions in poor urban areas. Its innovative model, known as “business unusual”, invites local governments to self-select to join the initiative, contribute their own funds and obtain support for capacity–building through a coaching of coaches model to effectively implement impactful, scalable, and sustainable FP/AYSRH programs. Over 50 local governments across East Africa—in Kenya, Tanzania, and Uganda—have partnered with TCI to scale up FP/AYSRH high-impact interventions. A total of 1,367 public and 729 private health facilities, including 150 pharmacies, are receiving financial and technical support from TCI in East Africa. In Kenya, TCI is currently implemented in eight counties, namely Kilifi, Kericho, Migori, Uasin-Gishu, Mombasa, Nairobi, Nyamira, and Vihiga. However, private sector engagement through pharmacies was piloted in 3 counties, specifically Kilifi, Mombasa, and Nairobi. TCI aimed at increasing the capacity of pharmacies to offer quality family planning at the community level and develop a mechanism that would allow the pharmacists to record and showcase their client service data to ensure the visibility of the data, which would be important for the local government's decision-making.

As part of the private sector engagement, pharmacies were engaged through KPA, which has an expansive network of registered pharmacies in urban settings and can monitor operating standards. Between June 2019 and December 2020, 150 pharmacies (70 in Mombasa, 20 in Kilifi, and 60 in Nairobi) were identified and trained on the provision of FP services including referrals to the public sector per the current service provider guidelines. They were further registered and oriented on the use of TCI's online learning platform referred to as TCI University, and coached on the available Open Data Kit (ODK) tools for FP service data capture and reporting. The program increased the visibility of the pharmacies within the communities by branding them and supporting the distribution of information, education, and communication (IEC) materials, resulting in increased client flow.

## 3. Program implementation process

(a)
***County and pharmacy selection***

The 3 counties, Kilifi, Nairobi, and Mombasa, were jointly selected by TCI and the KPA as the pilot counties based on two specific criteria; TCI had already established partnership with local governments while KPA had a presence with a high number of operating pharmacies in the three areas. KPA member pharmacies were selected in the 3 counties based on the following criteria: 1) legally registered by both county and professional boards; 2) selling and providing FP commodities; 3) member of KPA; 4) location within or serving an urban slum population; 5) owners of the pharmacy present and managing the business; 6) willing to keep and share FP records with the public health system; 7) owners having smartphones that could be used for reporting onto the ODK platform; and 8) open to monitoring and support visits to be conducted by KPA, MoH, and TCI. A call for participation in the initiative was sent out to pharmacies in the three counties. A total of 150 pharmacies were selected to participate based on these criteria.

(b) ***TCI's Linkage with the Ministry of Health***

TCI provided logistical support to Kenya's MoH through coaching and dissemination of appropriate FP and AYSRH guidelines to the 150 participating pharmacies. During the coaching sessions, the pharmacists were introduced to their respective MoH focal persons for coordination of supportive supervision, mentorship, and further coaching based on gaps identified. The KPA representatives participated in monthly county program implementation team meetings led by the county MoH where they discussed implementation progress and identified and addressed arising challenges. Linkages between the pharmacies and public health facilities to enable the process of client referral to nearby public health facilities were also clarified and effected from the start of the initiative

(c) ***Training on data capture and submission through the mobile Open Data Kit (ODK) platform and on the TCI dashboard***

TCI trained the KPA members on its program implementation model, reporting templates, and systems. In Kenya, the pharmacy data is not submitted to the government's MoH HMIS reporting platform and analysis of pharmacy contribution is only dependent on periodic surveys. To facilitate reporting and data visibility, therefore, the TCI project developed and trained the pharmacists on documentation and reporting of service uptake *via* the ODK system and on the navigation of performance results on the TCI dashboard. The KPA members and implementing pharmacies were provided with access to the TCI dashboard to facilitate navigation and utilization of data.

(d) ***Capacity-building of pharmacy attendants***

Capacity-building workshops were conducted with the initial training of 20 master coaches over three days having been selected with support from KPA. The training focused on the provision of quality contraceptive services using the current MoH FP service provider guidelines. County reproductive health coordinators and certified pharmacists served as workshop facilitators. The master coaches in turn supported the step-down training and coaching within their respective counties. During the step-down training and coaching, the pharmacists were oriented on the provision of FP methods, with an emphasis on youth-friendly contraceptive services. Several adult-learning principles were deployed, including group discussions, demonstration, return demonstration (participants demonstrating back to the trainers what had just been demonstrated to them), and role-play on quality FP counseling. The coaching content included materials on counseling clients to support informed choice, data capture, and referrals to nearby public health facilities for other methods and/or management of potential side effects of contraceptive use. Though pharmacists have a medical services background, these sessions served to orientate and disseminate the revised Kenya National FP guidelines at community pharmacy outlets. The training was based on the following resource materials: Kenya MoH's family planning service providers guidelines (2018**)**, World Health Organization's Family Planning: A Global Handbook for Providers, the TCI-EA FP provider checklists, and TCI University resources.

(e) ***Provision of FP products and referrals for long-acting reversible contraceptives***

The participating pharmacies were able to stock and offer a range of contraceptive methods including condoms, Oral Contraceptive Pills (OCP), Emergency Contraceptive Pills (EC), and injectable contraceptives as per the Kenya FP policy and guideline of 2016. They referred those that wanted Long-Acting Reversible Contraceptives (LARCs) and permanent methods to nearby public health facilities since they are not allowed to offer them, although they counseled and sold commodities to those who needed them. These products are purchased from their supplier at no subsidy since they are a profit-making entity.

(f) ***Branding of the pharmacies and demand generation***

TCI supported the pharmacies to increase demand for their services. Community health volunteers (CHVs) were linked to the pharmacies and conducted community mobilization while distributing IEC materials such as leaflets on myths and misconceptions about FP as well as highlighting the availability of FP services in public facilities and participating pharmacies in their locations. The pharmacy's staff were also issued with branded T-shirts and caps that bore FP messages.

(g) ***Quality control and monitoring***

Joint supportive supervision was conducted with KPA, MoH, and TCI. This supervision was crucial in addressing implementation challenges and ensured a successful program outcome. MoH provided additional coaching where competency gaps existed. KPA also participated in the program implementation team meetings led by the public health management teams, where they reviewed their performance and prioritized the next steps for action. These meetings helped to strengthen the PPP relationship through continued dialogue with MoH. Data quality audits were conducted to monitor the accuracy, completeness, and timeliness of reporting. The service performance statistics were shared by the pharmacists with MoH and KPA by the 5th of every succeeding month.

(h) ***Regular collection of service uptake data***

FP provision data was captured and reported using the mobile ODK application. Primary data was collected using black books at the community pharmacy outlets. The respective county health management teams, KPA officials, and TCI staff regularly validated the data reports and monitored them for accuracy and completeness. The information collected included the name of the pharmacy where a client was served, date of service delivery, client sex, client referral source, the client visit type (new or revisit), FP method received, and client age. Data from the black books were entered onto digitized forms on ODK and submitted to a centralized server routinely. Data was summarized and analyzed on a monthly basis and shared by KPA with the MoH during the PIT meetings. Qualitative data was also collected from observational field visits and other TCI knowledge-sharing mechanisms and forums.

## 4. Discussion

### 4.1. Outcomes of the pilot program

(a)
***Increased FP service uptake***

FP service data from all participating pharmacies were analyzed by method for all visits to generate total visits by method for a given month period. To convert this to *family planning client volume*, twelve-month moving sums were applied to the total visits by method to account for seasonality in the data (see Table 1 and Data source*: TCI Program Management Information System* Table 2). Afterward, Couple-Years of Protection (CYP) indices were applied to the 12-month moving sums to adjust for revisits. The client volume for each method was summed up by period to estimate the number of clients visiting pharmacies within a given 12-month period. A total of 150 pharmacies reported this FP uptake data between October 2019 and December 2020.

**Table 1:** Pharmacy service data (October 2019-December 2020).

County	Number of pharmacies included in this PPP	Reporting rates	Total Contraceptive Client visits	Total male client visits	Total female client visits	Referrals to health facilities
Kilifi	40	13%	1,135	384	751	2
Mombasa	50	22%	11,959	2,861	9,098	97
Nairobi	60	43%	30,538	9,333	21,205	228
Total	150	27%	43,632	12,578	31,054	327

TCI Program Management Information System.

**Table 2:** Annual contraceptive client volume by age group and period (October 2019-December 2020).

Age group	Oct 2019–Sep 2020 (12 months) (*n* = 2,314)	Nov 2019–Oct 2020 (12 months) (*n* = 2,418)	Dec 2019–Nov 2020 (12 Months) (*n* = 2,629)	Jan 2020–Dec 2020 (12 Months) (*n* = 2, 821)	Total
<15 Years	11	12	13	13	49
15–19 Years	69	72	79	84	304
20–24 Years	512	530	573	603	2218
>24 Years	1,722	1,804	1,964	2,121	7611
Total	2,314	2,418	2,629	2,821	10182

TCI Program Management Information System.

A total of 43,632 FP client visits were recorded—71% of the visits were by female clients while 29% were by male clients. Adjusting for couple years of protection and seasonality, this translates to about 2,821 annual FP client volume within the latest twelve-month period of which 48% obtained an injectable, 25% an oral pill 24% an emergency contraceptive pill, and 3% a condom. The majority of clients (75%) were older than 24 years, 21% were 20–24 years, 3% were 15–19 years, and 1% were <15 years.

The 2016 revision of the Kenya National FP guidelines now allow pharmacies to administer both subcutaneous and intramuscular DMPA. This has enabled the expansion of access to include the various age groups of clients and the data correlates with other service uptake data which shows injectable as the most frequently reported method among users (see Table 3) (1).

(b) ***Improved referrals and linkages***

**Table 3:** Annual contraceptive client volume and methods (October 2019 to December 2020).

Geography	Method	Oct 2019—Sep 2020 (12 Months)	Nov 2019–Oct 2020 (12 Months)	Dec 2019–Nov 2020 (12 Months)	Jan 2020–Dec 2020 (12 Months)
Kilifi	Condom	2	2	2	2
	Emergency contraceptives (ECP)	22	22	23	25
	Injectable	21	22	23	28
	Pill	12	12	13	14
	Total	57	58	61	69
Mombasa	Condom	17	18	19	21
	ECP	173	181	194	220
	Injectable	221	228	246	279
	Pill	180	187	202	229
	Total	591	614	661	749
Nairobi	Condom	63	65	70	72
	ECP	375	388	420	431
	Injectable	843	890	980	1,049
	Pill	386	403	438	453
	Total	1,667	1,746	1,908	2,005
Total	Condom	82	85	91	95
	ECP	570	591	637	676
	Injectable	1,085	1,140	1,249	1,356
	Pill	578	602	653	696
	Total	2,315	2,418	2,630	2,823

TCI Program Management Information System.

Referrals and linkages between the private and public sectors are essential for any effective FP program for quality and continuity among women seeking FP methods that are not available within the private sector. As a result of the initiative, there was evidence of referrals and subsequent linkages between the private sector provider and public health facilities for purposes of continuity to service that were unavailable at the community pharmacy. From the program data, all the 43,632 clients were offered counseling services at the pharmacies. Out of the counseled clients, 327 (0.75%) were referred to public health facilities for other FP methods that were not being offered/available at the pharmacies, especially the LARCs. A small number (399) of these clients were only counseled.

(c) ***Improved FP data visibility from community pharmacies***

The pilot had 150 pharmacies with a focus on increasing access to quality FP services and strengthening public-private partnerships for a more inclusive FP program. The community pharmacies' FP service statistics submitted routinely through the ODK app and visualized in the TCI dashboard played a key role in this initiative, which sought to improve the support data-driven decision making.

Twenty-seven percent (27%) of the pharmacies submitted reports on uptake between October 2019 and December 2020 across the three counties. Within the same period, a total of 43,632 FP client visits were recorded. The 150 pharmacies represent 2% of the registered pharmacies that are offering FP services in Kenya.

(d) ***Strengthened capacity of pharmacy to offer quality FP services***

The capacity for staff of 150 pharmacists was built to provide an expanded method mix of FP commodities and information. Consequently, 43,632 client visits were recorded, clients were either counseled only, counseled and referred, or counseled and provided with FP commodities. This demonstrates aspects of quality measurements in FP service provision within the pharmacies. Joint supervision was conducted between MoH and the pharmacies where the quality of care issues were identified, including poor reporting. Coaching was then provided to pharmacy staff for the provision of quality services to clients and reporting of service statistic data using the ODK system to increase the visibility of their family planning data and use it for planning, specifically restocking family planning commodities.

(e) ***Strengthened collaboration between MoH and the private sector***

There was a strengthened collaboration between MoH and community pharmacies because of the TCI engagement in the 3 counties. Program data showed that in Mombasa and Nairobi, the pharmacy representatives in the region were able to participate in the monthly program implementation meetings held jointly with the MoH staff to share and discuss their performance and get an opportunity for further coaching on the weak areas. The coaching was informed by the gaps identified during support supervision and service statistic data. In Mombasa for instance, infection prevention was discussed during this meeting which facilitated the provision of safety boxes to the community pharmacy staff and the eventual incineration of the filled-up safety boxes in linked public health facilities at no additional cost. During the monthly program implementation meeting, the pharmacies were allowed to share their results with the MoH team. This provided an opportunity for visibility of the contribution they make to the national grid.

### 4.2. Lessons learned

There is need to create incentives to motivate pharmacies when implementing the interventions including awarding continuing professional development (CPD) points based on participation in capacity strengthening activities for scale to more locations.

Working with professional organizations like the Kenya Pharmaceutical Association (KPA) provides adequate capacity–building support to the pharmacy staff, especially regarding data capture/reporting processes. Pharmacies are a key entry point for increased uptake of FP beyond the adolescent and youth. It promotes access to contraception among males in the urban set up who may be less willing to go to the public health facilities which are predominantly used by the women for their FP needs.

TCI EA also applied a capacity-building approach of using peers to coach one another on the implementation. This approach is flexible taking into consideration that the pharmacies are profit-making entities that utilize local government staff, who are trained on the TCI model and high-impact interventions. This approach to coaching facilitated rapid knowledge and capacity transfer to pharmacies on service provision, data capture and reporting in the outlets' real-time. It is possible to capture the service statics data from the pharmacies if they are coached and oriented on data capture dashboard where they are able to support real-time reporting of service statics data and increase its visibility.

TCIs pharmacy engagement strategy provides a unique framework for policymakers and different stakeholders on public-private sector engagement in the delivery of FP services in urban settings.

## 5. Constraints/limitations of the approach

1. Given that this TCI intervention was implemented in select sites in Nairobi, Mombasa, and Kilifi Counties, the findings reported in this paper may not be representative of the whole country.
2. Given that pharmacies are profit-making entities, the intention to promote voluntary informed-choice FP may not be fully understood or appreciated. In addition, reporting rates varied across pharmacies and periods. In some months, these were below 30%. The pharmacies cited a lack of motivation to report.
3. The program did not collect baseline data (i.e., before the start of the PPP program in the 3 counties) to determine the exact change in modern contraceptive uptake and reporting rates. The program dashboard has enabled the analysis of service statistics data at the pharmacy level.

## 6. Conclusion

Community pharmacies play an important role in complementing the resource-limited public health system in improving access to family planning, especially in serving women with unmet needs for contraception. Although data reporting is a challenge for pharmacists, the intervention still reached women, men, and adolescents with contraceptive services and referrals to health facilities for additional FP services beyond the scope of the pharmacies, reflecting this sector's importance. Using a flexible yet structured mode of capacity–building is critical for improving access to an expanded method mix within the private sector, thus increasing the ability of the pharmacies to meet the FP demand in the community. There is a need for collaborative efforts on the part of MoH and other stakeholders to assess the implementation of policies guiding the engagement of provision of FP in pharmacies and including quality of FP service provision, data capture, and reporting processes. The poor reporting suggests a need for alternative approaches to motivate the pharmacies to report in government reporting systems. Future research should focus on understanding how to strengthen pharmacy data capture, visibility, and use.

## Data Availability

The original contributions presented in the study are included in the article/Supplementary Material, further inquiries can be directed to the corresponding author.
